# Effect of Fluid Resuscitation Strategies for Obese Patients with Sepsis and Septic Shock: A Systematic Review

**DOI:** 10.1007/s44231-022-00019-y

**Published:** 2022-10-27

**Authors:** Yijun Zhang, Minjie Wang, Zongqing Lu, Min Yang

**Affiliations:** 1 The Laboratory of Cardiopulmonary Resuscitation and Critical Care Medicine, The Second Affiliated Hospital of Anhui Medical University, Hefei, China; 2 The Second Department of Intensive Care Unit, The Second Affiliated Hospital of Anhui Medical University, Hefei, 230601 Anhui China

**Keywords:** Obesity, Sepsis, Initial fluid resuscitation

## Abstract

**Purpose:**

As the Surviving Sepsis Campaign (2021) recommended, patients with sepsis should be given a liquid infusion of 30 ml/kg (ideal body weight). However, the strategy may result in insufficient resuscitation for obese patients with sepsis. Therefore, we conducted a systematic evaluation of the effectiveness of the initial resuscitation strategy in obese sepsis patients.

**Materials and methods:**

A computer search of PubMed, Embase, Cochrane library, and other databases collected cohort studies from the beginning of the survey to December 2021 to include articles evaluating initial resuscitation strategies for sepsis-obese patients.

**Results:**

Of the six studies included, five used ideal body weight infusion strategies, and three used actual body weight infusion strategies. Differences in fluid volume were observed between the two strategies, but no significant difference was observed in the mortality of obese sepsis patients. In addition, there may be an infusion strategy other than the above two infusion methods, and the safety and efficacy of the new infusion strategy are unclear. The obesity paradox has been observed in most infusion strategies.

**Conclusion:**

The association between obesity and infusion strategy has rarely been investigated in patients with sepsis and septic shock, and the existing results are conflicting. The risk of bias in all included studies was moderate or high. Before providing broad recommendations on the optimal first resuscitation approach to lower the chance of mortality, further clinical trials, and prospective research need to be done.

## Introduction

Over the past decades, the global prevalence of overweight and obesity increased steadily with the improvement of living standards and becoming a public health problem. According to the latest epidemiological studies, more than one-fifth (20.6%) of adolescents and one-third (38.9%) of adults suffer from obesity in the United States [1, 2], while the prevalence of overweight, general obesity, and abdominal obesity were up to 38.80, 13.99, and 43.15% respectively in China [3]. It is well known that obesity is closely associated with the occurrence of diabetes, hyperlipidemia, cardiovascular and cerebrovascular diseases [4]. The previous studies have demonstrated that obese patients were closely related to a higher incidence of surgical site infection [5], postpartum infection [6], or even COVID-19 [7]. In critical care settings, treating obese people has long been a concern.

The early fluid resuscitation strategy improves prognosis and reduces organ failure and mortality risk in patients with septic shock [8–10]. Despite SSC 2021(Surviving Sepsis Campaign) recommendations for standardized initial fluid resuscitation (30 ml/kg) [8], a few studies have shown that obese patients received lower relative fluid volume than non-obese patients [11]. The differences could originate from the infusion strategy. Determining whether the discrepancy will affect the prognosis of sepsis obese patients remains an open question. In addition, although it is known that obesity harms severe patients, there are many conflicting findings regarding the protection of obesity in patients with sepsis. This phenomenon is clinically known as the “obesity paradox” (lower mortality in obese people). The molecular mechanism underlying this paradox remains to be elucidated but likely results from different clinical factors and therapeutic interventions. It remains to be seen whether this phenomenon can be observed with the same infusion strategy.

Therefore, we conducted a systematic evaluation to explore the optimal fluid infusion strategy during initial resuscitation in obese patients with sepsis and whether the “obesity paradox” could be observed under the same infusion strategy.

## Methods

### Eligibility Criteria

The review question we tried to answer was: Does the initial infusion strategy according to SSC guidelines reduce mortality in obese sepsis patients? It was formulated in accordance with the PICOS (population, intervention, comparison, outcomes, and study type) criteria (Table 1). Studies were selected on the basis of the following criteria: (1) Observational studies or randomized controlled trials related to the use of crystalline or colloidal fluids for initial resuscitation in patients with obese sepsis or obese septic shock. (2) These studies classified the sepsis patients according to the basis of WHO (World Health Organization) obesity classification criteria; (3) IBW infusion strategy and non-IBW infusion strategy were used as the main comparison content. (4) Primary outcome measures report all-cause mortality or hemodynamics during hospitalization. Secondary outcome measures included length of ICU stay, endotracheal intubation, and other measures. We excluded reviews, letters, correspondence, editorials, and nonhuman studies, but the reference lists of these articles were searched to identify other potential studies.

**Table 1:** PICOS (Population, Intervention, Comparison, Outcome and Study type) criteria for the inclusion of studies

Parameter	Inclusion criteria
Population	Obese patients with sepsis or septic shock
Intervention	Ideal weight infusion strategy
Comparison	Non-ideal weight infusion strategy
Outcome	All-cause mortality, hemodynamics during hospitalization
Study type	Controlled trial and observational studies

### Information Sources

The following electronic databases were assessed, covering studies published until December 2021: PubMed, Embase, Cochrane Library, OVID, China National Knowledge Infrastructure (CNKI), and Wanfang Database. No date or language restriction was used predefined while conducting this search.

### Search Strategy

The terms ‘obesity’ and ‘sepsis’ were obtained during this search. No terms referring to interventions or controls were used to amplify our search strategy. In December 2021, database searches were performed using the terms ‘overweight’ or ‘obesity’ and ‘sepsis’ or ‘Pyemia’ and related terms to obtain the broadest possible results. Potentially eligible papers were searched by screening reference lists and grey literature.

### Study Selection

Yijun Zhang and Minjie Wang searched independently, according to predefined inclusion and exclusion criteria. Duplicate literature deletion, title, and abstract screening for relevance were done using NoteExpress software (3.5.0.9054). Then, the full text was acquired to determine inclusion eligibility eventually. Disagreements were resolved by consensus-based discussion or by a third reviewer’s opinion.

### Data Extraction and Quality Assessment

Two reviewers (Yijun Zhang and Minjie wang) independently extracted data from each study using the extract table template. The extracted data were as follows: author information, year of publication, study type, BMI categories studied, sample size, the definition of sepsis, and outcomes. The primary outcome was the mortality of obese sepsis patients. Secondary outcomes included hospital length of stay and hemodynamic change. A third reviewer (Zongqing Lu) assessed all the studies for the completeness of their data extraction.

### Data Synthesis and Analysis

Given the significant methodological and statistical differences between studies, combining data using meta-analysis techniques were considered inappropriate. This article reviews the literature on the effect of initial fluid resuscitation infusion on mortality in obese and overweight sepsis patients. A synthesis of narratives is presented.

## Results

### Literature Search

**Fig. 1:**
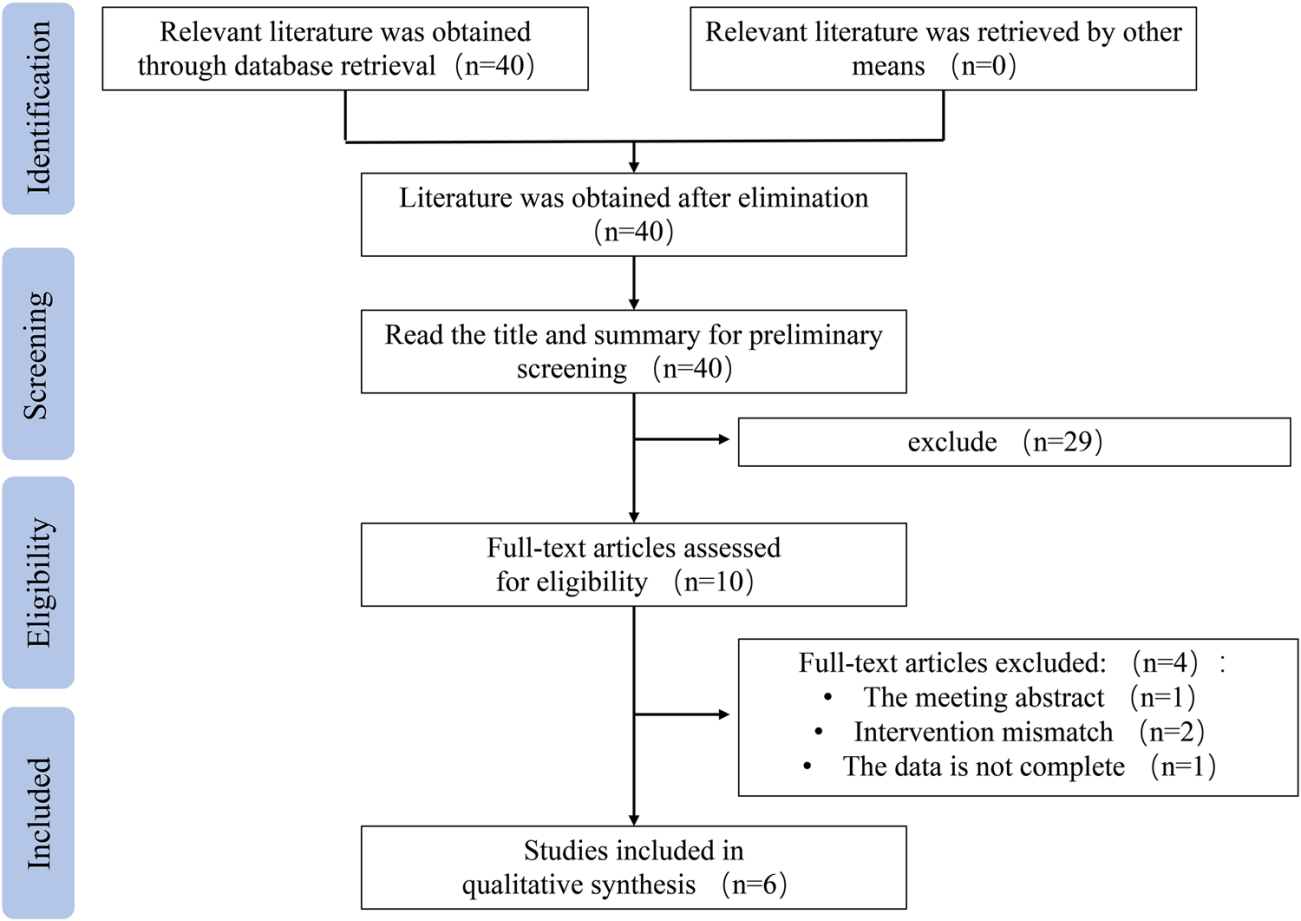
Study flow diagram for study selection

Overall, we identified 40 records and included six original articles after eliminating duplicate and unqualified headlines. Figure 1 shows a detailed flow chart of the screening process.

### General Characteristics of the Studies

From the time of publication, four of the included studies were published in the last 3 years. Geographically, four studies were performed in America and one in Europe. A multicenter study involving the United States, Canada, Saudi, and Arabia. All subjects were age 18 or older. The majority of studies were retrospective, and only one study was prospective. The primary characteristics of the six included studies are described in Table 2.

**Table 2:** Characteristics of studies evaluating effectiveness of initial fluid resuscitation strategies for obese patients combined with sepsis and septic shock

First author, year, country	Study type	BMI categories studied	Sample size	Definition of sepsis	Weight basis for fluid replacement	Type of fluid, dose, time	Outcome
Taylor 2017 American	Retrospective	Underweight: < 18.5,	4157	As code sepsis defined as	IBW	Liquid crystal,	Hospital mortality
	Cohort study,	Normal: 18.5–24.9,		A suspected infection with	ABW	30 ml/kg,	ICU mortality
	Multicenter	Overweight:25.0–29.9,		either refractory hypotension	AdjBW	3 h	Hospital length of stay
	Cohort	Obese:30.0–39.9,		or an initial lactate level greater than or equal to			ICU length of stay
		Very obese: ≥ 40		4 mmol/L			
Kaseer 2021 American	Retrospective	Obese: ≥ 30.0	72	Sepsis plus end-organ	IBW	NR,	Hospital mortality
	Cohort study,			Dysfunction, or lactate level	Non-IBW	30 ml/kg,	Hospital length of stay
	Single center			Greater than 2 mmol/L		NR	ICU length of stay
							Composite of progression
							to septic shock
							Persistent hypotension
							Initiation of vasopressors
Ward 2020 American	Retrospective	Underweight: < 18.5,	1032	Sepsis 2.0	IBW	Liquid crystal,	Mortality
	Cohort study,	Normal: 18.5–24.9,			ABW	30 ml/kg,	ICU Admit
	Single center	Obese: ≥ 30.0				3 h	ICU length of stay
							Intubation
							Delayed hypotension
Antal 2019 Romanian	Prospective	Normal: 18.5–24.9,	71	The 2012 SSC definitions	AdjBW	Crystalline or	Blood pressure
	Cohort study,	Overweight:25.0–29.9,		Sepsis 3.0	IBW	Colloidal liquid,	Cardiac output
	Single center	Obese:30.0–39.9,				< 10 ml/kg	Stroke volume
		Very obese: ≥ 40				10–19.99 ml/kg	Systemic vascular resistance
						20-29 ml/kg	Capillary refill time
						> 30 ml/kg,	
						3 h	
Arabi 2013 Canada,	Retrospective	Underweight: < 18.5,	2882	Sepsis 1.0	ABW	Crystalline or	Hospital mortality
American, Saudi Arabia	Cohort study,	Normal: 18.5–24.9,				colloidal liquid,	ICU mortality
	Multicenter	Overweight:25.0–29.9,				NR,	ICU length of stay
	Cohort	Obese:30.0–39.9,				6 h	Hospital length of stay
		Very obese: ≥ 40					
Kuttab 2019 American	Retrospective	Obese: ≥ 30.0	1032	Sepsis 2.0	IBW	Liquid crystal,	Mortality intubation
	Cohort study,					30 ml/kg,	Delayed hypotension
	Single center					3 h	ICU admit
							ICU length of stay

*NR* not reported, *ICU* intensive care unit, *SSC* surviving sepsis campaign, *BMI* body mass index, *IBW* ideal body weight, *ABW* actual body weight, *AdjBW* adjusted body weight

### IBW Infusion Strategy

Of these, five studies used the IBW (ideal body weight) infusion strategy, with case fatality rates that ranged from 8 to 30.9%. After adjusting for population, condition, and treatment-related variables, Taylor et al. [12] found that the mortality rate of obese patients using the IBW infusion strategy was 16.1%. Ward and Kuttab et al. observed similar results [13, 14]. In the study of Antal, only 49.3% of patients met SSC requirements when the initial resuscitation dose was administered in the first 3 h according to local guidelines, while fluid volume increased significantly in each group after subsequent dose adjustment, and all-cause mortality remained at 30.9% [15]. Kaseer et al. [16] found that severe sepsis progressed to septic shock in 18% of patients using the IBW infusion strategy and without the use of vasopressor. The above patients were reported as obese, with a mortality rate of 8%.

### ABW Infusion Strategy

ABW (actual body weight) infusion strategy was used in three studies, and the mortality associated with this infusion strategy varied widely. Taylor and Ward et al. found 8.4 and 12% mortality rates in obese sepsis patients who used ABW infusion strategies [12, 13]. Arabi et al. conducted a nested cohort study of septic shock patients at 28 medical centers in Canada, the United States, and Saudi Arabia between 1996 and 2008. They found that mortality rates reached 50% among obese and morbidly obese patients enrolled [17].

### Other Strategies

Taylor et al. [12] adjusted the initial resuscitation fluid volume according to the recommended drug dose, and the fluid volume was between IBW and ABW. The mortality rate of the population using this infusion strategy was 15.3%.

### Obesity Paradox

The retrospective analyses of an extensive American multicenter database reported by Taylor et al. reported. After adjusting for population, condition, and treatment-related variables, there was no significant difference in mortality between obese and non-obese patients with sepsis [12]. In another study, compared with regular patients, obese patients with septic shock had lower crude mortality (odds ratio (OR) 0.80, 95% confidence interval (95% CI) 0.66–0.97). Mortality was not statistically significant between the two groups after combining sepsis intervention and baseline characteristic multivariate logistic regression analysis [17]. However, Kuttab et al. discussed sepsis obesity in their subgroup and showed that the mortality rate of obese patients was lower than that of non-obese sepsis patients [14]. Most studies have observed an “obesity paradox” phenomenon, irrespective of differential baseline levels of obese patients.

### Risk of Bias Assessment and Grade Profile Evidence

The details about the risk of bias were respectively shown in Table 3. ROBINS-I scale was used to evaluate literature quality. Two studies were classified as having a high risk of bias for included cohort studies. Kaseer et al. [16] could not determine whether the baseline results were reliable because they did not specify the infusion method or duration. Meanwhile, Kuttab et al. [14] did not report fluid data for obese patients, so we categorize them as high risk through discussion.

**Table 3:** The risk of bias assessment of included cohort studies by using the ROBINS-I tool

Study/domain	Confouding	Selection of participants into the study	Classification of interventions	Deviations from intended interventions	Missing data	Measurement of outcomes	Bias in selection of the reported result	Overall risk
Taylor 2017	Moderate	Low	Low	Low	Low	Moderate	Moderate	Moderate
Kaseer 2021	Moderate	Low	Moderate	Low	Low	Low	Moderate	High
Ward 2020	Moderate	Low	Low	Low	Low	Low	Moderate	Moderate
Antal 2019	Moderate	Low	Moderate	Moderate	Low	Moderate	Low	High
Arabi 2013	Moderate	Low	Moderate	Low	Low	Low	Low	Moderate
Kuttab 2019	Moderate	Low	Low	Low	Low	Moderate	Low	Moderate

*ROBINS-I* risk of bias in non-randomised studies—of interventions

## Discussion

This systematic review of observational studies assessed the effect in initial resuscitation effects of ABW compared to IBW in obese patients with sepsis. A total of six studies were included, and the results varied widely across studies, making it impossible to make a solid conclusion. The results of observational studies are subject to different factors and must be interpreted cautiously. In all studies, the risk of bias is moderate to high.

### Survival Benefits of IBW and ABW

The SSC 2021 guidelines recommend using at least 30 mL/kg (IBW) of crystalloid during initial fluid resuscitation. This initial recovery of fixed volume is based on observational evidence [18]. In the PROCESS [19], ARISE [20], and PROMISE [21] trials, the average amount of fluid received before randomization was also in the 30 mL/kg range, leading the guidelines to conclude that such infusion doses were generally accepted in the clinic [22].

However, the guidelines of the SSC do not deal with the obese population. It is worth noting that the obese population adopted distinct infusion strategies that may differ both in IBW and ABW. For different infusion strategies, the amount of fluid obtained could vary greatly. In an observational study of fluid resuscitation in burn patients, the fluid volume of the obese patients with ABW was compared to that of the normal-weight patients, showing a significant reduction. Moreover, obese patients received more fluid after adjusting the infusion strategy to IBW [23]. Arabi et al. [17] exclusively investigated different intervention results of obese sepsis patients and found that the obese and morbidly obese groups received fewer fluids in the initial resuscitation stage. Many studies [15–17, 24] have mentioned the difference in fluid infusion between obese and non-obese patients. In the first 12 h after confirmation of severe sepsis, the non-IBW group received a higher amount of fluid, with 13% of patients requiring mechanical ventilation initiation versus 2% in the IBW group. This difference may be due to pulmonary edema caused by excessive resuscitation. Additionally, fluid boluses could lead to a positive fluid balance and excess fluid in the interstitial space [25, 26], resulting in tissue edema, decreased oxygen delivery, and increased mortality [27–29]. The effect of this fluid difference is the source of debate over IBW and ABW infusion strategies.

After comparing IBW and non-IBW infusion strategies in obese sepsis patients, Kaseer et al. found that mortality was 8% in the IBW group and 4% in the non-IBW group [16]. On the contrary, Antal et al. found that patients’ hemodynamic indicators improved after adjusting the infusion strategy to IBW [15]. We reason that these seemingly conflictive results might be due to the following reasons. The minimal small sample size in some studies may have contributed to the result. Complications may occur around initial fluid recovery, and these factors were not considered in current studies. Another possibility is that patients with fewer physical disorders themselves receive less fluid than regular patients. However, from a clinical point of view, the risk of fluid insufficiency in initial resuscitation is significant. The SSC recommended infusion dose may not be the optimal infusion strategy, but there is no evidence of higher quality that has shown that ABW is more effective than IBW. In addition, Taylor et al. [12] propose a new management strategy and found the method has potential survival benefits in obese patients. The adjusted body weight dose for obese patients will mean a lower fluid volume than the traditional actual body weight dose but a higher fluid volume than the ideal body weight dose. The strategy showed a survival benefit of fluid administration based on weight adjustment compared with the ideal weight administration strategy (OR 0.29, 95% CI 0.11–0.79). This suggests that better infusion strategies may exist for obese sepsis patients. However, the safety and effectiveness of this infusion strategy are still unknown and need to be validated.

Future studies or guidelines need to define more significant differences in the amount of fluid needed for resuscitation in different populations and establish methods for assessing the volume needs of obese patients, taking into account differences in body mass index in these patients.

### Is there an“Obesity Paradox” in Obese Sepsis Patients?

At least 25% of adults admitted to ICU in a developed country have an overweight, obese, or morbidly obese body mass index. Although there is much evidence that obesity reduces overall life expectancy, most observational studies show that absolute mortality is 5–15% lower in obese patients than in normal-weight patients [30]. Higher metabolic levels, activation of the renin-angiotensin system, and the inability of adipose tissue to produce immunomodulatory molecules may account for the survival advantage [31]. There is much evidence that overweight and obesity may have a protective effect on patients with sepsis, but morbid obesity is not included [32–35]. Better ICU survival has been observed in overweight/obese patients compared to those who are underweight, have a normal weight, or are morbidly obese.

The “obesity paradox” results remain inconsistent and contradictory in our systematic review. Although the balance of the infusion strategy baseline has been controlled, there may be several reasons to account for this discrepancy. The leading causes may be methodological problems, including selection bias in patients with sepsis, analysis of uncontrolled confounding factors such as smoking, potential site, type of infection, nutritional status, treatment style, race, etc. In addition, the small sample size is also a factor.

### Future Directions

The limitations of the current literature suggest the need for studies to provide a high level of evidence on infusion strategies and doses for initial resuscitation in obese patients with septic shock and to clarify their status in treating obesity in this particular population. Given the growing number of obese people and the specificity of this population, evaluation of infusion strategies, comorbidities, and costs in normal-weight patients cannot be assumed to apply to obese people.

## Conclusions

In patients with sepsis and septic shock, the relationship between obesity and infusion strategy has only occasionally been researched, and the available data are contradictory. The risk of bias in all included studies was moderate or high. In conclusion, more clinical trials and prospective studies should be conducted before making general recommendations about the best initial resuscitation strategy to reduce the risk of death.

## Data Availability

Not applicable.

## Abbreviations

NR: Not reported
ICU: Intensive care unit
SSC: Surviving sepsis campaign
BMI: Body mass index
IBW: Ideal body weight
ABW: Actual body weight
AdjBW: Adjusted body weight
